# Behavioral Immune System Responses to Coronavirus: A Reinforcement Sensitivity Theory Explanation of Conformity, Warmth Toward Others and Attitudes Toward Lockdown

**DOI:** 10.3389/fpsyg.2020.566237

**Published:** 2020-11-26

**Authors:** Alison M. Bacon, Philip J. Corr

**Affiliations:** ^1^School of Psychology, University of Plymouth, Plymouth, United Kingdom; ^2^Department of Psychology, City, University of London, London, United Kingdom

**Keywords:** conformity, personality, reinforcement sensitivity theory (RST), perceived vulnerability to disease (PVD), behavioral immune system, COVID-19, coronavirus

## Abstract

Behavioral immune system (BIS) describes psychological mechanisms that detect cues to infectious pathogens in the immediate environment, trigger disease-relevant responses and facilitate behavioral avoidance/escape. BIS activation elicits a perceived vulnerability to disease (PVD) which can result in conformity with social norms. However, a response to superficial cues can result in aversive responses to people that pose no actual threat, leading to an aversion to unfamiliar others, and likelihood of prejudice. Pathogen-neutralizing behaviors, therefore, have implications for social interaction as well as illness behaviors and responses to health communications. In this study, we investigate how PVD influences conformity, attitudes to other people and to lockdown regulations through the lens of the Reinforcement Sensitivity Theory (RST). RST describes personality in terms of biologically-driven approach and avoidance motivations which support personal goals. Participants from the United Kingdom public (*N* = 605) completed an RST personality questionnaire and then read either (a) coronavirus morbidity-mortality statistics and current United Kingdom government lifestyle regulations, (b) just the regulations (as presented in most government publicity materials), or (c) no information at all. They all completed the Perceived Vulnerability to Disease scale to assess BIS-relevant Germ Aversion and Perceived Infectability, followed by questions measuring social conformity, warmth toward others and attitudes toward lockdown measures. Significantly lower PVD scores were observed in the no-information condition, with the other conditions showing no difference. In terms of RST, approach behaviors related to goal-drive persistence work alongside fear in explaining conformity to social norms. Reward related approach behaviors partially explained warmth toward others, indicating that social rewards gained through interaction continue to be strong drivers of behavior. We found no role for RST traits in attitudes toward lockdown. Overall, coronavirus-related behavior is not driven purely by fear, but also by social and/or protection goals regulated by approach motivation. This study presents new insights into public perceptions of coronavirus and government regulated lifestyle restrictions, helping to explain social behaviors in terms of biologically driven mechanisms. Such understanding is vital if we are to successfully motivate public behavior to constrain spread of the virus.

## Introduction

The outbreak and rapid spread of the Coronavirus Disease (COVID-19) has presented critical challenges for individuals and society. Across the world, governments have imposed lifestyle restrictions, limiting physical contact between people in an attempt to slow the rate of infection; and an important aspect has been the requirement for those showing symptoms to self-isolate for 14 days (World Health Organization, 2020). While these measures have served to protect lives and public health resources, the absence of a vaccine and regular media coverage of a mounting death toll has contributed to a sense of, sometimes severe, anxiety (Garfin et al., 2020). Alongside these psychological outcomes, the constraints of living under what is commonly known as “lockdown” (leaving home only infrequently and for very specific and essential reasons) has resulted in stressful life circumstances (Droit-Volet et al., 2020; Kang et al., 2020; Rubin and Wessely, 2020), serving to further exacerbate the general anxiety about the pandemic.

However, anxiety may have some beneficial aspects. For example, Harper et al. (2020) showed that, relatedly, fear of coronavirus predicts compliance with government lockdown regulations and positive behavior change, such as social distancing and increased hand washing. Fear was found to be more important in this respect than personal moral values around fairness or protecting the vulnerable. Harper et al. (2020) discussed what they term “functional fear”: certain negative emotions are actually normative and adaptive rather than pathological, and they may have evolved as protective function to keep us safe. Similarly, substantial evidence suggests that a primary evolved disgust response underpins behaviors in situations such as the current pandemic, with a set of unconscious psychological responses acting as a first line of defense against potential pathogens. This evolved defense has been termed the *behavioral immune system* (Schaller and Park, 2011; Schaller, 2015; Murray and Schaller, 2016). This system is of particular interest in the context of coronavirus because it is related to triggering a sense of vulnerability to infectious disease which, in turn, has been linked to increases in conforming behaviors and attitudes (Murray and Schaller, 2012) – an imperative if government regulations are to be effective.

Schaller and Park (2011) describe how the behavioral immune system evolved as a reaction to significant species threat presented by infectious diseases. While we, like other species, developed a physiological system for combatting disease, the mounting of an immune response is costly to the organism in terms of energy that could otherwise be deployed in maintaining other vital physical and behavioral systems. Immune responses, such as a raised temperature, fever and fatigue, are debilitating which, in evolutionary terms, reduces opportunity for species to sustain vital activities, such as food gathering, childcare and reproduction. Furthermore, the physiological immune response is *reactive*, coming too late in terms of prevention, as it does not activate until the body is already infected. This leaves a ‘window of opportunity’ for the disease to take hold and damage the body, sometimes beyond repair. Accordingly, the evolution of a *proactive* psychologically based motivational system, which can facilitate behavioral avoidance of infection, is clearly adaptive (Murray and Schaller, 2016). In developing the most widely used measure of PVD, Duncan et al. (2009) established two subfactors, both specific to infectious diseases. Germ Aversion predicts responses rooted in intuitive emotional appraisals of risk, whereas Perceived Infectability predicts responses informed by more rational cognitive appraisals. The distinction is consistent with evidence that Germ Aversion more strongly predicts implicit negative associations toward individuals with visible differences (Park et al., 2003, 2007), whereas Perceived Infectability more strongly predicts implicit negative associations with individuals regarded as potentially immunocompromised (Duncan and Schaller, 2009).

An important marker of BIS sensitivity is disgust (Oaten et al., 2009). Although this may not seem immediately relevant to the coronavirus context, it is important to note that it is not only evoked by exposure to repugnant physical stimuli, but can be experienced as a sense of distress and revulsion in any context which connotes disease or potential contamination (Taylor, 2019). For instance in terms of coronavirus, the public are recommended to wash their hands frequently and guidelines emphasize how the virus can remain alive on surfaces touched by an infected person. The very thought of touching an object in a public place can be enough to elicit a disgust response in some individuals. During the 2009 swine flu epidemic, disgust sensitivity predicted fear of acquiring influenza (Wheaton et al., 2012; Brand et al., 2013) and in the avian flu epidemic of 2005, PVD (specifically germ aversion) was found to relate to specific fears about contracting that disease (Green et al., 2010). However, germ aversion is not isomorphic with disgust sensitivity. Whereas disgust sensitivity measures assess emotional responses to a broad range of potentially disgust-arousing circumstances, germ aversion is specific to situations connoting the potential transmission of infectious diseases (Duncan et al., 2009).

Fearful behavioral immune system responses can influence many social-cognitive phenomena, including face recognition, social categorization, stereotype activation, conformity to majority opinion, political ideology, and memory (Griskevicius et al., 2006; Schaller et al., 2007; Miller et al., 2010; Murray and Schaller, 2016; Fernandes et al., 2017; Tybur et al., 2016). Historically, adherence to social norms has served to protect against disease (e.g., hygiene behaviors); and research evidence indicates that perceived vulnerability to disease is associated with the endorsement of statements such as “Breaking social norms can have harmful, unintended consequences,” as well as to actual behavioral conformity (Murray and Schaller, 2012). Recent evidence from the US suggests that people have already become more socially conservative during the coronavirus pandemic (Rosenfeld and Tomiyama, 2020). This finding is in accord with another key result from Murray and Schaller’s (2012) research, namely that individuals who perceive themselves as highly susceptible tend to express greater liking for people described as having personality traits indicative of greater conformity (e.g., “conventional” and “traditional”). Importantly, however, harsh judgments made in the context of perceived vulnerability are made only when the object is perceived to have deviated (or has the potential to) from social norms which offer protection against disease transmission (Horberg et al., 2009; Murray and Schaller, 2012). In the present context, this could be a response to violations of social distancing or stay-at-home rules, although it may also result in stigmatization of groups that are heuristically associated with disease, whether or not they actually present a threat (Park et al., 2003, 2007; Miller and Maner, 2012).

The current pandemic is reported to have started in China, and there have been many reports of xenophobia against individuals perceived to be of Chinese or Asian ethnicity (BBC, 2020; Devakumar et al., 2020; Rzymski and Nowicki, 2020; Tabri et al., 2020). Overall, the behavioral immune system may have important implications for social behaviors and relationships in the context of the present pandemic. In the present study, we are particularly interested in how perceived disease threat and related self-protection motives influence conformity with Government restrictions and negative responses to other people. We examine individual differences in these responses through the lens of the Reinforcement Sensitivity theory of personality.

### Reinforcement Sensitivity Theory

We investigated individual differences in behavioral immune system influenced perceived vulnerability to coronavirus through the lens of the Reinforcement Sensitivity Theory (RST) of personality. RST is a useful perspective in this context as, like the behavioral immune system, it explains motivated behavior linked to environmental cues. RST assumes that personality is underpinned by biologically driven systems of approach and avoidance motivation, and their conflict (Gray and McNaughton, 2000). Approach/avoidance motivational tendencies drive attention to social and environmental cues, manifesting in characteristic patterns of cognition and behavior. RST is widely recognized, in conceptual and psychometric terms, to represent valid personality traits of widespread application (Corr et al., 2013).

Reinforcement Sensitivity Theory comprises a set of motivational systems which explain individual differences. The *behavioral approach system* is sensitive to appetitive stimuli and motivated goal-directed approach behaviors (Gray and McNaughton, 2000). The primary function of this system is to move the organism along a spatio-temporal gradient toward a final biological reinforcer via a number of distinct but related processes: *Reward Interest* and *Goal-Drive persistence* characterize the early stages of approach, and can be distinguished from *Reward Reactivity* and *Impulsivity*, which become active as the desired outcome becomes immediate and attainable. Activation of the behavioral approach system leads to the experience of hope, excitement, drive to achieve, and elation when goals are attained (Corr and Cooper, 2016).

Krupić et al. (2016b) investigated the relationships between behavioral approach factors and motives underpinning two groups of evolved resource acquisition behaviors: competition (e.g., stealing, trickery, aggression) and cooperation (e.g., social exchange, altruism). Reward Interest was associated with a tendency to explore the environment in search of reward (resources/relationships) and with caring and reciprocity, both with family and wider community. Goal-Drive Persistence was associated with social exchange and cooperation over a longer term, while individuals high in Reward Reactivity showed a tendency to threat avoidance, maintaining safety and demonstrating commitment to relationships with close others. While all three factors are associated with prosociality, the approach motivations behind them differ, attaining a social reward, behaving cooperatively and maintaining that relationship by negating threat. Impulsivity, however, although also an approach factor, was associated with competiveness and a tendency to perceive the self as superior to others. In the present context, we can imagine that people with cooperative prosocial traits will wish to follow government guidelines and maintain social norms, not just for their own safety, but for that of their immediate family and the wider community. Individuals higher in Impulsivity may be less likely to do so, because of a sense of insuperability as well as the tendency to act without thinking of the consequences.

Reinforcement sensitivity theory defines two further systems concerned with defensive behaviors. The *Fight-Flight-Freeze System* is associated with fear and mediates reactions to aversive stimuli, leading to active avoidance and escape behaviors. The *Behavioral Inhibition System* is activated by goal conflict, which occurs when there is activation of both the Fight-Flight-Freeze System and Behavioral Approach System (Gray and McNaughton, 2000; Perkins et al., 2007; Corr, 2011; Corr and Cooper, 2016; for review, Corr and Cooper, 2016). This system is related to passive avoidance, behavioral caution, and enhanced vigilance and arousal. We can imagine how a dispositionally fearful or cautious individual may experience high levels of behavioral immune system activation in the pandemic situation.

Despite the potential to explain intentional and actual behaviors, there has been very little health-related research on RST. One recent study examined pandemic-related behavior. Bacon and Corr (2020) showed that concerns about coronavirus relate to higher levels of both approach related Reward Reactivity and the Fight-Flight-Freeze System. These findings point to the presence of fear but also an urge to take action, resulting in psychological conflict. Bacon and Corr (2020) suggested that proactive behaviors, such as buying and hoarding household items, may be an behavioral approach tactic which supports the goal of retaining a sense of normality – these products are available when needed even if the individual is choosing to self-isolate (not compulsory at the time of the study) thus resolving the conflict to some degree. Also relevant to the current research is evidence that RST personality traits influence the perception of health-related persuasive communications. The Behavioral Inhibition System’s emotions (anxiety and emotional conflict) make individuals more receptive to loss messages, while emotions related to the Behavioral Approach System (including anger) are more receptive to gain messages (Yang et al., 2012). Understanding more about how RST influences pandemic-related behavior may have implications for lifestyle advice directed at combatting spread of the virus.

Limited research has examined individual differences in behavioral immune system activation and perceived vulnerability to disease in terms of personality and the work which has been conducted has focused on the Big Five model. The available research indicates that both openness to experience (i.e., curiosity and willingness to try new things) and extraversion (i.e., sociability and gregariousness) are negatively associated with perceived vulnerability to disease (Schaller and Murray, 2008; Duncan et al., 2009). It has been suggested that activation of the behavioral immune system suppresses gregariousness and desire for social interaction, for the obvious reason that individuals who have more social contacts are at higher risk of infection (Nettle, 2005; Schaller and Murray, 2008; Mortensen et al., 2010; Murray and Schaller, 2016). Our RST approach is not at odds with these findings. The curiosity and desire for novelty typical of openness to experience relates to behavioral approach system activation, particularly Reward Interest, and is negatively associated with activation in the Fight-Flight-Freeze System, but not the Behavioral Inhibition System (Corr and Cooper, 2016). Openness to experience, therefore, is about exploration of the new without fear (Corr and Krupić, 2017). The social reward sensitivity of Extraversion is also associated with behavioral approach system, including the Impulsivity aspect (Corr et al., 2013).

### The Present Study

The present study takes a novel approach to understanding how personality affects perceived vulnerability to disease (PVD) specifically in the context of the coronavirus pandemic. Our overall aim is to establish that RST personality traits can play a role in individual differences in PVD and in associated attitudes toward conformity and lockdown and feelings of warmth toward other people. In setting out our initial predictions, we made no distinction between the germ aversion (GA) and perceived infectability (PI) aspects of PVD. First, we aimed to activate the behavioral immune system by asking participants to read information about the pandemic, and then measuring the levels of PVD they report. We presented three groups of participants with one of three information conditions: (a) no information; (b) details of the UK Government’s stay at home regulations, with which most people are already familiar; or (c) this information plus morbidity and mortality statistics (as current at the time of data collection). Based on previous research using similar methods (for a review, see Tybur et al., 2014) we expected that condition 2 would lead to higher levels of PVD (as indexed by questionnaire scores) compared to condition 1 (the control group). In condition 3, we expected that the statistics would place the regulations into context, making them more salient and, as a result, lead to even higher PVD scores **(*Prediction 1*).**

Secondly, we predicted a positive association between perceived vulnerability and Fight-Flight-Freeze in all conditions, reflecting fear of contagion. If personality is a driver of individual differences we would expect fight-flight-freeze to account for variance in PVD over and above the effect of condition **(*Prediction 2*)**.

Previous research has shown PVD to be positively associated with self-reported conformity, negatively associated with warmth toward other people, and positively with favorable attitudes toward lockdown. We examined the extent to which RST accounted for variance in these three outcome variables over and above effects of PVD. For attitudes toward conformity, we predicted that fear, and hence Fight-Fright-Freeze, would explain variance over and above that accounted for by PVD **(*Prediction 3***). For warmth, we expected that social-reward sensitive approach factors (reward interest, goal-drive persistence, and reward reactivity) would explain variance independently of PVD. In addition, given Bacon and Corr’s (2020) finding that people seem to be experiencing goal conflict between wanting to stay safe and retain a normal lifestyle, we also expected to observe effects of the Behavioral Inhibition System as this system mediates conflict between approach inclinations and fear (***Prediction 4***). Similarly, for attitudes to lockdown, we again expected fight-flight-freeze and the behavioral inhibition system to present effects (***Prediction 5***).

## Materials and Methods

### Participants

Six hundred and five members of the UK public (173 Male, 426 female, 6 other; *M_age_* = 32.78, *SD* = 1.64) were recruited through Prolific, an online research recruitment platform – data from such sources is more representative of the general population than samples recruited directly (Woods et al., 2015). Socio-economic status (SES) was assessed by the MacArthur Ladder Scale, which ranks self-reported social class on a ladder with 10 rungs (Adler et al., 2000) – the higher rungs represent individuals who have more money, education, and prestigious jobs. The mean report was 5.40 (*SD* = 1.64) with 52 people (13.4%) placing themselves on the bottom three rungs and 40 (6.3%) on the top three rungs. Five hundred and thirty participants (87.6%) identified as White, 16 (2.6%) as Black, 31 (5.1%) as Asian, 21 (3.5%) as mixed race and 7 (1.2%) as other. The majority of participants were educated to A’ level (212, 35%) or degree (227, 37.5%) level. Fifty-nine (9.8%) reported having masters level education and 11 (1.8%) having a PhD/doctorate. Ninety-two (15.2%) reported GCSE level qualifications and 4 (0.7%) reported no formal qualifications.

Participants were randomly allocated to one of three conditions: Condition 1 – *N* = 202, *M_age_* = 32.67, *SD* = 11.54; 59 males, 142 females, 1 other; Condition 2 – *N* = 202, *M*_age_ = 33.28, *SD* = 11.46, 49 males, 151 females and 2 other; Condition 3 – *N* = 201, *Mage* = 32.39, *SD* = 11.95; 65 males, 133 females and 3 other. The groups did not differ significantly on age, *F*(2,603) = 0.31, *p* = 0.74. Chi square tests of independence confirmed the other demographic variables were randomly distributed throughout the three groups (*p* > 0.2 in very case).

### Procedures and Materials

The study was conducted online. On accessing the study, participants were first given information about it and provided informed consent by checking a box before the study could begin. They then completed the following measures.

*Reinforcement Sensitivity Theory of Personality Questionnaire* (RST-PQ; Corr and Cooper, 2016) is a 65-item questionnaire yielding scores on RST traits. Behavioral Approach System (BAS) factors: Reward Interest (RI; 7 items, e.g., “I am very open to new experiences in life”) Goal-Drive Persistence (GDP; 7 items, e.g., “I put in a big effort to accomplish important goals in my life”); Reward Reactivity (RR; 10 items, e.g., “Sometimes even little things in life can give me great pleasure”); Impulsivity (I; 8 items, e.g., “I often do risky things without thinking of the consequences”). Behavioral Inhibition System (BIS; 23 items, e.g., “I’m always weighing-up the risk of bad things happening in my life”); and the Fight-Flight-Freeze System (FFFS; 10 items, e.g., “There are some things that I simply cannot go near”). Participants respond on a scale from 1 (not at all) to 4 (highly) and mean responses are calculated to generate a score for each subscale. All scales showed good reliability in our sample: RI α = 0.82; GDP α = 0.89; RR α = 0.80, impulsivity α = 0.75, Behavioral inhibition system α = 0.94; FFFS α = 0.78.

*Generalized Anxiety Disorder* (GAD-7; Spitzer et al., 2006) is a 7-item self-administered questionnaire used as a screening tool and severity measure for generalized anxiety. Participants are asked how often in they have experiences a series of problems such as Feeling nervous, anxious or on edge over the previous 2 weeks. They respond on scale from 0 (not at all) to 3 (nearly every day). Overall score is derived as mean of all 7 responses. In our sample, reliability was very high (α = 0.91).

*Patient Health Questionnaire* (PHQ-9; Spitzer et al., 2006) presents the same instructions and response scale as the GAD-7 but assesses levels of depression across nine items such as *Little interest or pleasure in doing things*. Mean responses are calculated to give an overall score. Reliability was very good in the present sample: α = 0.87. Anxiety and depression were not a key focus of this study, but these measures were included as covariates. Depression is associated with immune responses and may have evolved as a way of keeping an unwell individual from close socialization with others (Raison and Miller, 2017). GAD is associated with poor health and related health anxiety, which is found to influence the aspects of PVD related to perceived vulnerability, but not germ aversion (Duncan et al., 2009).

At this point, we presented participants with information about coronavirus in order trigger PVD. We manipulated the level of coronavirus-relevant information across conditions. In Condition 1, they were simply told *This questionnaire is about your health*.

In Condition 2, they were told:

This questionnaire is about your health. Please read the following information first and then answer the questions below:

Because of the current Coronavirus (COVID-19) outbreak, the Government have given instructions to everyone in the United Kingdom about what they can and cannot do. The instructions tell us to:

• Stay at home
• Only go outside for food, health reasons or work (but only if you cannot work from home)
• If you go out, stay 2 m (6ft) away from other people at all times
• Wash your hands as soon as you get home
• Do not meet others, even friends or family. You can spread the virus even if you don’t have symptoms.

In condition 3:

This questionnaire is about your health. Please read the following information first and then answer the questions below:

We are currently experiencing a worldwide pandemic caused by the coronavirus (COVID-19). Worldwide, nearly 3 million people have been infected and over 200,000 have died to date. In the United Kingdom, we have over 150,000 confirmed cases and over 20,000 people have died.

This information was then followed by the Government guideline information as presented to Condition 2. The morbidity and mortality statistics were correct at time of the study and sourced from Public Health England (2020).

**FIGURE 1:**
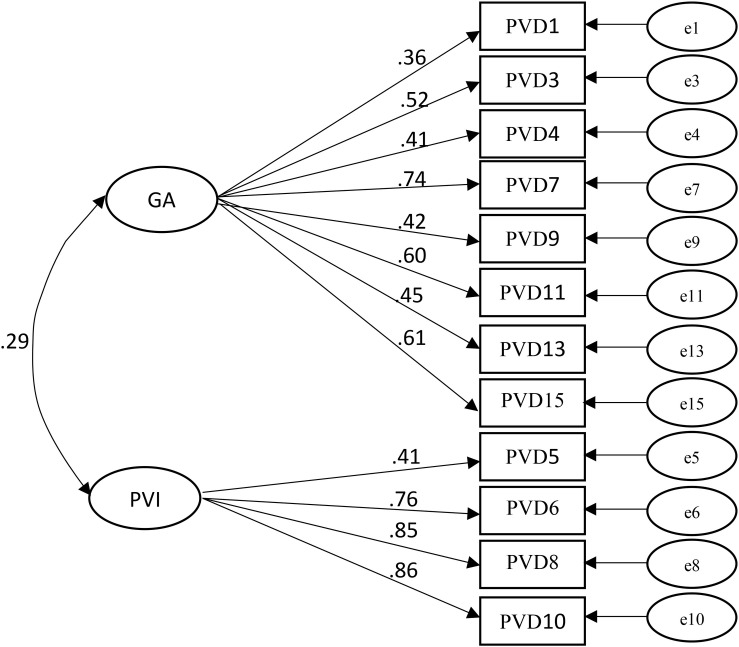
Standardized loadings of PVDQ items on GA and PI in the final version of the scale used for analysis in present study. All paths significant at *p* < 0.001.

After reading the above information, all participants completed the *Perceived vulnerability to disease scale* (PVDS: Duncan et al., 2009). This 15-item measure assesses behavioral immune system activation across two subscales: Perceived Infectability (PI; 7-items, e.g., “If an illness is ‘going around’, I will get it”) and Germ Aversion [GA; 8 items, e.g., “It does not make me anxious to be around sick people” (reverse scored)]. Responses on a scale from 1 (Strongly disagree) to 7 (strongly agree) are averaged to obtain subscale scores. Díaz et al. (2016) have highlighted that reliabilities are often lower for GA than PI, and they also review research which has questioned the factor structure of the PVDS. They conclude that a 2-factor structure is appropriate but were required to remove two items from analysis in order to achieve an acceptable fit to their data. In the present study, an exploratory factor analysis was conducted in SPSS v24 with maximum likelihood estimation and promax rotation. A forced two-factor solution accounted for 41.69% variance overall (11.50% PI; 30.19% GA). Results suggested that all PVDQ items loaded on the expected factors apart from one (item 2, *If there is an illness going around I will get it*) which loaded similarly on both GA (β = 0.61) and PI (β = 0.56). However, examination of the scree plot suggested the presence of three factors with Eigenvalues greater than 1, so we ran the analysis again forcing a three-factor solution which accounted for 48.50% variance overall. GA loading remained as previously, while the PI scale spilt into two factors, one accounting for 12% variance, and the other 6.40%. This latter factor loaded on just three PVD items, 5, 12, and 14. We then performed confirmatory factor analyses using SPSS AMOS v25. The models are shown in Figure 1 and fit indices in Table 1.

**TABLE 1:** Results of SEM of perceived vulnerability to disease (PVDQ) data.

	**Model 1 Three factors (without item 2)**	**Model 2 Two-factors (without items, 2, 5,12, 14)**
χ^2^ (df), *p*	260.20 (64), *p* < 0.001	134.14 (43), *p* < 0.001
RMSEA (90% CI)	0.07 (0.06,0.07)	0.06 (0.05,0.07)
CFI	0.93	0.95
SRMR	0.05	0.05

Firstly, we fitted the three-factor model (Model 1 in Table 1) leaving out item 2. As Table 1 shows, the Chi-square statistic was significant, but other fit indices were acceptable. Although items 5, 12, and 14 load separately to the other PI items, they all clearly relate to the PI construct (item 5, *My past experiences make me believe I am not likely to get sick even when my friends are sick*; item 12, *I am unlikely to catch a cold, flu or other illness, even if it is “going around*”; and item 14, *My immune system protects me from most illnesses that other people get*”). Interestingly these are the only three reverse scored items on the PI subscale. Whether that has led to some anomaly in responding is unclear and a more detailed psychometric examination of the PVD scale is beyond the scope of the present article. We, therefore, omitted these three items from analysis and fitted a two-factor (GA and PI) model (Model 2 in Table 1). Chi-square was again significant but all other indices suggested a good fit. Cronbach’s alpha statistics for these final scales indicated acceptable reliability, GA α = 0.74, PI α = 0.81. Based on our structural equation model (Figure 1) we imputed standardized GA and PI scores from AMOS v25. These scores control for error variance and were used in all further analyses.

*Conformity, warmth toward other people and attitudes toward lockdown*: Participants completed a 10-item scale developed by the authors. They were presented with the following instructions: *Please indicate how much you agree or disagree with each of the following 10 statements in terms of how you have been thinking and feeling over recent weeks. There are no right or wrong answers. Some questions refer to lockdown. This term refers to the current measures to combat coronavirus where everyone is asked to stay at home except for essential reasons.* We presented comprised 4 items measuring conformity (e.g., *Breaking social norms of behavior can have harmful unintended consequences)*, three measuring attitudes to others (e.g., *I generally feel warm toward other people, even those I don’t know well*) and three measuring general attitudes toward lockdown (e.g., *I think the lockdown is a helpful measure in combatting the coronavirus*). Participants responded on a scale from 1 (strongly disagree) to 5 (strongly agree) and mean scores were calculated for each subscale. In line with Murray and Schaller (2012) who used a similar procedure, we conducted a confirmatory factor analysis on our 10 questions which yielded a clear three-factor solution with acceptable fit indices: CFI = 0.91, RMSEA = 0.08 and SRMR = 0.06. Cronbach’s alpha was adequate for self-reported conformity (α = 0.70) and negative attitudes to others (α = 0.69), though low for attitudes to lockdown (α = 0.58). Average inter-item correlations were moderate, though significant (Conformity 0.33; attitudes to others 0.43; attitudes to lockdown 0.33). All 10 questions can be found in our supplementary materials http://www.philipcorr.net/includes/asp/download_file.asp?id=456.

## Results

Our dataset is available at http://www.philipcorr.net/includes/asp/download_file.asp?id=453. Table 2 presents descriptive statistics for all key measures. GA and PI we present the imputed score derived from our structural equation model as described previously.

**TABLE 2:** Descriptive statistics.

	**Condition 1**		**Condition 2**		**Condition 3**		**Full Sample**	
**Measure**	**Mean**	***SD***	**Mean**	***SD***	**Mean**	***SD***	**Mean**	***SD***
GA	1.50	0.44	1.70	0.41	1.63	0.44	1.61	0.44
PI	2.54	1.23	2.53	1.24	2.58	1.12	2.55	1.20
RI	2.37	0.66	2.41	0.61	2.42	0.60	2.40	0.62
GDP	2.66	0.72	2.71	0.69	2.70	0.64	2.69	0.69
RR	2.63	0.53	2.72	0.51	2.69	0.50	2.69	0.51
IMP	2.29	0.59	2.36	0.58	2.31	0.55	2.32	0.57
BIS	2.50	0.68	2.49	0.64	2.48	0.69	2.50	0.65
FFFS	2.41	0.64	2.52	0.64	2.41	0.61	2.45	0.63
Conformity	3.77	0.54	3.77	0.52	3.79	0.56	3.78	0.54
Warmth toward others	3.62	0.85	3.61	0.79	3.58	0.82	3.60	0.82
Positive attitude to lockdown	3.98	0.77	3.91	0.78	3.99	0.77	3.96	0.77

*GA, germ aversion; PI, perceived infectability; RI, reward interest; GDP, goal-drive persistence; RR, reward reactivity; IMP, impulsivity; BIS, behavioral inhibition system; FFFS, fight-flight-freeze system.*

### Prediction 1

The three conditions differed significantly in germ aversion (GA), *F*(2,604) = 11.76, *p* < *0.001*, and *post hoc* tests with Bonferroni correction indicated that Condition 1 scored significantly lower than the other two conditions, but that conditions 2 and 3 did not differ significantly from one another (*p* = 0.29). No significant differences between conditions was observed for perceived infectability (PI), *F*(2,604) = 0.09, *p* = 0.91. This indicated that our manipulation was effective in eliciting PVD in terms of GA. Although the different levels of detail given in conditions two and three did not result in differences between those two groups, both were higher in GA than the group given no information. The three conditions did not differ on Conformity, warmth toward other people or attitudes to lockdown (*p* > 0.5 in all cases).

### Prediction 2

Prediction 2 stated that RST Fight-flight-freeze scores would be positively associated with PVD and account for variance over and above that explained by condition. Table 3 presents correlations between our key outcome measures (GA, PI, conformity, warmth toward others and positive attitudes to lockdown) and RST trait scores. We computed Bonferroni corrections for these analyses which resulted in a *p*-value of 0.001, and correlations are indicated as significant at this level. Across all three conditions, fight-fight-freeze is significantly and positively association with GA, and with PI in conditions 2 and 3, those where PVD was primed with coronavirus related information.

**TABLE 3:** Correlations between measures for each of the three conditions.

		**PI**	**Conform**	**Warm**	**Ldown**	**RI**	**GDP**	**RR**	**IMP**	**BIS**	**FFFS**
1	GA	0.34*	0.14	−0.15	−0.01	0.04	0.08	0.11	0.03	0.13	0.30*
	PI		−0.06	−0.03	0.01	−0.03	0.01	−0.002	−0.02	0.17	0.15
	Conformity			0.07	0.04	0.04	0.11	0.22*	0.03	0.07	0.30*
	Warmth				0.11	0.28*	0.24*	0.24*	−0.01	−0.17*	−0.12
	Lockdown					−0.04	−0.033	−0.05	−0.13	−0.20*	−0.17
2	GA	0.32*	0.21	−0.12	0.18	0.05	0.18	0.10	0.02	0.07	0.36*
	PI		0.13	−0.15	0.03	−0.04	0.04	0.02	−0.07	0.16	0.24*
	Conformity			0.11	0.05	0.14	0.24*	0.13	0.03	−0.02	0.29*
	Warmth				−0.001	0.37*	0.25*	0.29*	0.17	−0.10	−0.01
	Lockdown					−0.04	0.08	0.14	0.01	−0.07	0.001
3	GA	0.36*	0.30*	−0.10	0.10	−0.02	0.10	0.16	0.08	0.11	0.27*
	PI		−0.002	−0.13	−0.02	−0.14	−0.10	−0.06	0.08	0.20	0.25*
	Conformity			0.16	0.05	−0.02	0.19	0.10	0.003	−0.02	0.17
	Warmth				−0.06	0.24*	0.33*	0.18	0.03	−0.16	−0.07
	Lockdown					0.12	0.07	−0.01	−0.16	−0.05	0.01

GA, germ aversion; PI, perceived infectability; RI, reward interest; GDP, goal-drive persistence; RR, reward reactivity; IMP, impulsivity; BIS, behavioral inhibition system; FFFS, fight-flight-freeze system. *sig. at 0.001.

In testing the second part of prediction 2, we computed multiple regression using the PROCESS macro for SPSS v.3.5, model 1 (Hayes, 2018). We entered Condition (Group 1 = −1, group 2 = 0, and group 3 = 1) and RST factors, together with sex (male = 1, female = 2), SES, age, educational level, ethnicity (White = 1, Others = 0), anxiety and depression as covariates. Our model accounted for 16% of variance in GA and suggested that older people, women, those of lower SES and those with Non-White ethnicity were most germ averse. A significant effect of condition, β = 0.16, *p* < 0.001, 95% CI [0.08,0.22] illustrated that participants who read coronavirus-related information prior to completing the PVD scale were more germ averse than those who read no information. A significant independent effect of fight-flight-freeze was also observed, β = 0.28, *p* < 0.001, 95% CI [0.13,0.25] supporting the second part of prediction 2. Although not specifically predicted, it is notable that we also observed significant main effects of goal-drive persistence, β = 0.14, *p* = 0.01, 95% CI [0.02,0.16], and the behavioral inhibition system, β = −0.13, *p* = 0.04, 95% CI [−0.16,−0.02]. No moderating effects of condition on the relationship between RST factors and GA were observed (*p range* = 0.54–0.98).

We conducted the same analysis on PI scores. The model accounted for 7% variance overall. Depression showed an independent effect (*p* = 0.01), but no significant effect of condition was observed (*p* = 0.93). Of the RST factors, only fight-flight-freeze presented a significant effect on PI, β = 0.18, *p* < 0.001, 95% CI [0.16,0.52]. No moderating effects were observed (*p* = 0.36).

Having established that RST traits were associated with PVD, we then examined the extent to which they could support conformity, warmth and attitudes to lockdown. In regression analyses, we entered the covariates as previously plus GA, PI and the RST trait scores. Table 4 presents the results for all three analyses.

**TABLE 4:** Results of regressions analyses on Conformity, Warmth towards others and attitude to lockdown.

	**Conformity Adj. *R*^2^ = 0.11**				**Warmth Adj. *R*^2^ = 0.22**				**Positive attitude to lockdown Adj. *R*^2^ = 0.06**			
	**95% CI**		**95% CI**		**95% CI**							
	**St. β**	***p***	**Lower**	**Upper**	**St. β**	***p***	**Lower**	**Upper**	**St. β**	***p***	**Lower**	**Upper**

Age	0.09	0.03	0.0003	0.01	0.004	0.92	−0.01	0.01	−0.01	0.82	−0.01	0.01
Sex	−0.04	0.30	−0.17	0.05	0.14	<0.001	0.11	0.37	0.07	0.11	−0.03	0.25
Education	−0.04	0.34	−0.08	0.03	0.13	0.001	0.05	0.18	0.03	0.44	−0.04	0.10
SES	0.05	0.23	−0.01	0.05	−0.01	0.84	−0.04	0.04	−0.04	0.35	−0.06	0.02
Ethnicity	0.01	0.90	−0.14	0.16	0.04	0.24	−0.07	0.29	0.13	0.002	0.11	0.48
Anxiety	0.02	0.80	−0.01	0.02	−0.04	0.50	−0.03	0.01	−0.14	0.06	−0.04	0.001
Depression	−0.09	0.21	−0.02	0.01	−0.00	0.97	−0.02	0.02	−0.16	0.04	−0.04	−0.001
Condition	−0.01	0.87	−0.07	0.06	−0.02	0.55	−0.09	0.05	−0.02	0.64	−0.09	0.06
GA	0.14	0.001	0.08	0.34	−0.13	0.002	−0.38	−0.09	0.12	0.01	0.06	0.37
PI	−0.04	0.32	−0.07	0.02	−0.04	0.30	−0.08	0.02	0.01	0.82	−0.05	0.06
RI	−0.09	0.12	−0.20	0.02	0.13	0.01	0.04	0.31	0.01	0.91	−0.13	0.15
GDP	0.14	0.01	0.03	0.24	0.09	0.11	−0.02	0.23	−0.01	0.89	−0.14	0.12
RR	0.07	0.19	−0.04	0.21	0.19	<0.001	0.15	0.45	0.07	0.19	−0.05	0.26
IMP	−0.02	0.64	−0.12	0.08	0.01	0.84	−0.11	0.13	−0.08	0.10	−0.23	0.02
BIS	−0.02	0.82	−0.14	0.11	−0.25	<0.001	−0.46	−0.16	0.10	0.16	−0.04	0.27
FFFS	0.23	0.001	0.14	0.34	0.01	0.78	−0.10	0.13	−0.07	0.19	−0.20	0.04

*PI, perceived vulnerability to infection; GA, germ aversion; RI, reward interest; GDP, goal-drive persistence; RR, reward reactivity; IMP, impulsivity; BIS, behavioral inhibition system; FFFS, fight-flight-freeze system.*

### Prediction 3

In terms of conformity, our model accounted for 11% variance with higher levels of GA presenting a significant effect. Independent variance was accounted for by activation of the fight-flight-freeze system as per Prediction 3, but also by goal-drive persistence.

**FIGURE 2:**
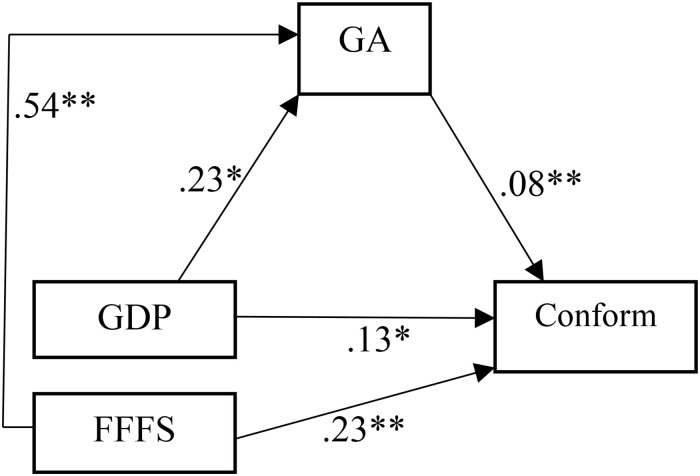
Mediating effects of germ aversion (GA) on the relationships between goal-drive persistence (GDP) and fight-flight-freeze (FFFS) on conformity. ^∗^Sig. at *p* ≤ 0.01; ^∗∗^Sig. at *p* ≤ 0.001.

We tested for mediating effects of GA on the relationship between both fight-flight-freeze and goal-drive on conformity using PROCESS v3.5. Model 4 (Hayes, 2018). Results are illustrated in Figure 2, left hand model. Both RST factors significantly accounted for conformity directly, but also indirectly via GA; goal-drive persistence β = 0.02, *95% CI* [0.001,0.04] and fight-flight-freeze β = 0.04, *95% CI* [0.01;0.07].

### Prediction 4

With warmth toward others as the dependent variable, regression with the same procedure produced a model accounting for 22% variance. GA accounted for variance negatively, but RST reward interest, reward reactivity and behavioral inhibition system also showed independent positive effects, in line with our prediction. However, no significant mediating effects of GA on the relationship between these RST factors and warmth was observed.

### Prediction 5

The same analysis on positive attitudes toward lockdown resulted in a model accounting for 6%, with a significant main effect of GA. However, no significant effects of RST were observed.

## Discussion

Despite the importance of personality in predicting everyday behavioral outcomes, there is very little available evidence about how it affects responses in pandemic or epidemic situations. The present study presented a novel approach to examining public responses to coronavirus and government behavioral guidelines in the United Kingdom. We focused on behaviors and attitudes predicted by the evolved behavioral immune system, a psychological first line of defense against infection, and explained these in terms of the RST of personality, which defines biologically driven approach and avoidance behaviors.

We activated behavioral immune system responses by presenting participants with information about coronavirus and required behaviors. We anticipated that those presented with morbidity and mortality statistics as well as a reminder of government behavioral regulations would report higher levels of PVD than those given just the regulations, or no information at all. The group who received no information prior to completing the PVD measure reported significantly lower levels of PVD but only in terms of GA. We found no difference between in the regulations-only and regulations + statistics conditions. Overall, this suggests our manipulation was effective at triggering the Behavioral Immune System in terms of germ aversion, but that the additional statistical information did not enhance the effect. The reason for this is unclear. It may be that participants were already very familiar with the government guidelines and simply disregarded the statistics, or that citing Global/National statistics was not sufficiently salient to affect PVD. Health information at a local community level is known to be more effective in communicating public health messages (Luck et al., 2006).

Our second prediction was that fight-fight-freeze would be related to PVD scores. This was, indeed, the case for both GA and PI, confirming that people who are naturally predisposed to fearfulness will generally show higher levels of PVD, as we might expect. However, we also found significant effects of goal-drive persistence and the behavioral inhibition system on GA (though not on PI). Goal-drive is part of the behavioral activation system in RST terms and therefore indicates a degree of proactive approach behavior, whilst the behavioral inhibition system deals with psychological conflict between these goals and fear (in this case of infection). Germ aversion represents discomfort within contexts where disease-causing germs might be transmitted. Congruent proactive goals may aim to prevent infection, such as by wearing mask or avoiding crowded places, however, such goals are not incongruent with fear and should not prompt behavioral inhibition system activation. Our results suggest that even individuals high in GA are experiencing degree of dissonance in their aversion.

Much prior research has suggested two key behavioral outcomes of PVD, conformity and a lack of warmth toward other people, particularly if they are perceived (rightly or wrongly) to carry a risk of infection. In the case of conformity, we observed an effect of Fight-Flight-Freeze, as expected, but also of Goal-Drive Persistence. However, conformity can be seen as a form of intentional action in pursuit of safety goals and, thus, an effect of goal-drive is congruent with fear of contagion. For warmth toward others, again in line with our prediction, we observed effects of RST approach factors reward interest and reward reactivity, together with behavioral inhibition system activation. Social goals are incompatible with fear of infection and, in mitigating this conflict, the behavioral-inhibition system may inhibit prepotent behaviors. However, if the social goal drive is strong enough (which it may be in individuals who are very prosocial, such as extraverts) some approach behavior will occur, tentatively, alongside risk-assessment (Gray and McNaughton, 2000; Corr and Krupić, 2017). In the RST behavioral activation system, reward interest is involved with identifying opportunities and wanting the rewards associated with them, goal-drive with planning and striving to fulfill the opportunity, impulsivity with actively grasping the rewards and reward reactivity with the positive emotional response which results (Corr and Cooper, 2016; Corr and Krupić, 2017). In the present study, the emotional aspects of Behavioral Approach System seem to influence warmth toward others, but not the proactive aspects. We suggest that the effects of reward interest and reactivity alongside GA reflect the desire for social rewards gained by friendliness toward others, despite feelings of aversion. This is not necessarily in contravention of social distancing rules, friendliness is often reciprocated without close contact (such as in a smile or saying hello) and this may be sufficient reward for many. The role of reward interest and reactivity in instances where people do break the lockdown rules is worthy of further study.

Finally, we conducted similar analyses in terms of attitudes toward lockdown regulations. We expected that support for the regulations would be positively associated with PVD and conformity, and therefore RST factors associated with conformity would play a role in supporting attitudes. However, we found a significant effect of GA only. It would seem that whatever the stresses and frustrations associated with lockdown, GA stimulates support for the restrictions as an effective measure in reducing spread of the virus, irrespective of personality or conformity with social norms in general. This would support further our suggestion above, that social rewards may be insufficient to break lockdown rules for most people.

The absence of psychological conflict (as evidenced by effects of the RST Behavioral Inhibition System) in the present study in terms of both conformity and attitudes to lockdown might appear to contradict the results of Bacon and Corr (2020); however, the differing results may arise from the time the two studies were carried out. Bacon and Corr’s data were collected at an early stage of the pandemic before lockdown and associated lifestyle restrictions were imposed in the United Kingdom. At that time, behaviors, such as panic buying and hoarding of food and household items, were widely reported and Bacon and Corr suggested that such behaviors were indicative of psychological conflict between the goal of living a normal life and fear about shortages amid a potential, but at the time very uncertain, lockdown. At the time of the present study, such behaviors had subsided. The fantasy of normality had become unsustainable and most people were resigned to, and actively engaged in, activity dictated by lockdown and social distancing regulations. Fear serves to move an individual away from potential contagion, and these avoidance behaviors also present proactive ways of staying safe.

Our results support recent data reported by Harper et al. (2020), who also emphasize the role of fear, albeit explained by different mechanisms. In their study, fear directly influenced protective behaviors such as hand-washing, but they present a caveat in that these behaviors are dictated by government policy and, therefore, may be a function of reluctance to deviate from this new normative social behavior, as much as they are explained by fear. Our results on conformity suggest that this may indeed be the case, but that fear is also implicated, as is the drive to achieve safety goals. Harper et al. did not measure conformity (hence their caveat) and we did not directly measure behavior. The two studies complement each other to show how fear can be one of the key drivers behind PVD, conformity and protective behaviors.

In this context, it worth noting how RST differentiates between fear and anxiety. Several recent papers (e.g., Droit-Volet et al., 2020; Garfin et al., 2020; Kang et al., 2020; Rubin and Wessely, 2020) discuss psychological effects of the pandemic in terms of anxiety. Anxiety (like worry) is future focused, it concerns thought about an uncertain future and what may, or may not, happen, and is linked to Behavioral Inhibition System. Fear, on the other hand is a response to an imminent threat linked to the Fight-Flight-Freeze system, which is responsible for triggering action to move the organism away from that threat (Gray and McNaughton, 2000; Corr and Cooper, 2016). A number of psychometric (Perkins and Corr, 2006; Krupić et al., 2016a), experimental (Perkins et al., 2007, 2012) and psychopathological (Bijttebier et al., 2009; Sylvers et al., 2011) studies have supported this differentiation. That we observed effects of fight-flight-freeze and not behavioral inhibition suggests that many people now perceive the threat of coronavirus as very real, and very imminent.

However, the Fight-Flight-Freeze System may not encapsulate all responses to immediate threat. In the face of an inescapable danger, we may not always have the opportunity to flee and freezing, unless we can successfully hide from the threat, may not be an effective way to protect ourselves. In this case, fight becomes the only option. However, a number of studies have found that measures of this type of defensive fight correlate negatively with fight-flight-freeze, and positively with behavioral activation (e.g., Harmon-Jones, 2003; Smits and Kuppens, 2005; Corr and Cooper, 2016). Corr and Cooper (2016) present a supplementary RST-PQ subscale to measure defensive fight, and Krupić et al. (2016c) have shown that defensive fight, together with the Reward Interest and Impulsivity aspects of the Behavioral Approach System, predicted tendencies to move toward a threat in dangerous situations. Conversely, behavioral inhibition, fight-flight-freeze and goal-drive persistence were associated with moving away from threat. Corr and Cooper (2016) suggested a problem with low base rates in response to their defensive-fight scale as, for most people, appropriate threat scenarios happen infrequently. However, contexts such as the coronavirus pandemic may present a rare opportunity to examine defensive–fight responses and further research should include a measure of this behavior.

Finally, it is notable how little effect of PI was observed in the present study. Our manipulation did not appear to elicit PI differentially across the three groups (as it did with GA) and their scores on PI were virtually identical. PI and GA were significantly correlated at a level consistent with previous literature (e.g., Duncan et al., 2009) and PI did present significant positive bivariate correlations with fight-fright-freeze in both conditions where we had primed the BIS. It also presented correlations with behavioral inhibition system activation though these did not quite reach significance once we had corrected for multiple analyses (Table 3). This suggests that PI may encompass aspects of both fear and anxiety. We included anxiety and depression as covariates in regression and depression did present an independent effect on PI, though otherwise these factors had relatively little effect in the presence of the other variables so it is unlikely that inclusion of the covariates suppressed effects of PI. In terms of RST, only FFFS significantly influenced PI in our regression analysis. In addition, PI showed no effect on any of our three outcome variables. A major public health threat will cause the behavioral immune system to be triggered in almost everyone to some extent (Taylor, 2019). It may be that participants were already feeling generally vulnerable to infection because of the publicity surrounding coronavirus. Germ aversion, however, may be a more context-specific emotion, evoked by a particular event or situation and therefore amenable to manipulation (in this case, by presentation of facts about the coronavirus). Germ aversion has been associated with context specific disease threat and during the avian flu epidemic of 2005, GA was found to relate to specific fears about contracting that disease (Green et al., 2010). Another explanation might be the nature of the PVD scale. While the GA subscale items fitted our data well, the PI ones did not. Indeed, we had to remove three PI items to find a model of PVD which adequately fitted our data. These were all reversed scored items and we included no attention checks in our test battery, though this does not appear to be a necessary requisite according to previous research. Díaz et al. (2016) discuss reported problems with reliability of the PVDQ subscales, though usually with the GA scale, and that, at the time of their article, only three published studies had utilized the two subscales separately, others having used a combined score. The PVD scale used in the present study was that originally proposed by Duncan et al. (2009) and is arguably the most widely used version. The PI subscale is concerned with subjective susceptibility to disease and the three items removed all refer to perceived immunological functioning in comparison to other people (perception that the respondent will not get a disease even if others do) it may be that this aspect of PI requires further psychometric investigation.

The study is not without limitations, including those inherent in self-report. We did not measure behavior directly and, although the factors we discuss are known to have behavioral consequents, we cannot categorically infer behavior from our results. Nor did we present standardized measures of conformity or warmth. Our approach was chosen in order to keep the questionnaire battery as short as possible in order to prevent fatigue, and there is precedent for our methods in Murray and Schaller (2012). Future studies might usefully attempt to replicate our results using standardized measures. Our data are cross-sectional in nature. Some of the differences between our results and those of Bacon and Corr (2020), mentioned above, illustrate how quickly the coronavirus situation, and associated social factors are changing. Most recently, and since our data was collected, the United Kingdom government have relaxed some aspects of lockdown and media reports are already suggesting public overreactions to this, with crowds flocking to parks and beaches making social-distancing unfeasible. There are suggestions that this may raise the probability of a second wave of the virus (e.g., Independent, 2020; The Guardian, 2020). Ongoing research should consider amendments to governmental policy and how social perception, and behavior, changes alongside this. Finally, this study was conducted very specifically within the context of coronavirus and the results may not translate to other conditions. It provides a useful platform on which to base research around other public health concerns such as seasonal flu, which leads to around 10,000 deaths each year in the United Kingdom. Important questions include attitudes to flu vaccinations given that under 50% of eligible adults with a long-term health condition took up the offer of a vaccination in 2019 (Public Health England, 2019).

## Conclusion

This study presents new insights into public perceptions of coronavirus and government regulated lifestyle restrictions, helping to explain social behaviors in terms of biologically driven mechanisms. Such understanding is vital if we are successfully to motivate public behavior to constrain spread of the virus. Our research also suggests that the level of behavioral information presented in government guidelines is appropriate to activate a perception of vulnerability, associated agreement with regulations and conformity. Importantly, we also identified that behavior is not driven purely by fear, but also by social and/or protection goals regulated by approach motivation. Previous research has suggested that the approach system is most receptive to gain messages in health communications (Yang et al., 2012). We, therefore, suggest that communication about coronavirus focus on the potential rewards of compliance at an individual level, as well as a national one. RST is a novel perspective from which to examine the behavioral immune system. Future research might examine further the intersection between BIS and RST, and how these two biologically driven systems can influence other health contexts where perceptions of vulnerability, and goal driven behaviors can have a substantial impact on wellbeing, both within the present pandemic situation, and beyond it.

## Data Availability Statement

The raw data supporting the conclusions of this article will be made available by the authors, without undue reservation.

## Ethics Statement

The studies involving human participants were reviewed and approved by the Faculty Research Ethics and Integrity Committee, Faculty of Health, University of Plymouth, United Kingdom. The patients/participants provided their written informed consent to participate in this study.

## Author Contributions

Both authors listed have made a substantial, direct and intellectual contribution to the work, and approved it for publication.

## Conflict of Interest

The authors declare that the research was conducted in the absence of any commercial or financial relationships that could be construed as a potential conflict of interest.
